# Simultaneous Near-Infrared Spectroscopy (NIRS) and Amplitude-Integrated Electroencephalography (aEEG): Dual Use of Brain Monitoring Techniques Improves Our Understanding of Physiology

**DOI:** 10.3389/fped.2019.00560

**Published:** 2020-01-21

**Authors:** Gabriel Fernando Todeschi Variane, Valerie Y. Chock, Alexandre Netto, Rafaela Fabri Rodrigues Pietrobom, Krisa Page Van Meurs

**Affiliations:** ^1^Grupo Santa Joana, Division of Neonatology, São Paulo, Brazil; ^2^Division of Neonatology, Department of Pediatrics, Irmandade da Santa Casa de Misericórdia de São Paulo, São Paulo, Brazil; ^3^Protecting Brains and Saving Futures Organization, São Paulo, Brazil; ^4^Division of Neonatal and Developmental Medicine, Stanford University School of Medicine and Packard Children's Hospital Stanford, Palo Alto, CA, United States; ^5^Neonatal Unit, Sociedade Beneficente Israelita Brasileira Hospital Albert Einstein, São Paulo, Brazil

**Keywords:** amplitude-integrated electroencephalography, near-infrared spectroscopy, neonate, neonatal intensive care, brain injury, neuromonitoring, neuroprotection

## Abstract

Continuous brain monitoring tools are increasingly being used in the neonatal intensive care unit (NICU) to assess brain function and cerebral oxygenation in neonates at high risk for brain injury. Near infrared spectroscopy (NIRS) is useful in critically ill neonates as a trend monitor to evaluate the balance between tissue oxygen delivery and consumption, providing cerebral and somatic oximetry values, and allowing earlier identification of abnormalities in hemodynamics and cerebral perfusion. Amplitude-integrated electroencephalography (aEEG) is a method for continuous monitoring of cerebral function at the bedside. Simultaneous use of both monitoring modalities may improve the understanding of alterations in hemodynamics and risk of cerebral injury. Several studies have described correlations between aEEG and NIRS monitoring, especially in infants with hypoxic-ischemic encephalopathy (HIE), but few describe the combined use of both monitoring techniques in a wider range of clinical scenarios. We review the use of NIRS and aEEG in neonates and describe four cases where abnormal NIRS values were immediately followed by changes in brain activity as seen on aEEG allowing the impact of a hemodynamic disturbance on the brain to be correlated with the changes in the aEEG background pattern. These four clinical scenarios demonstrate how simultaneous neuromonitoring with aEEG and NIRS provides important clinical information. We speculate that routine use of these combined monitoring modalities may become the future standard for neonatal neuromonitoring.

## Introduction

Continuous brain monitoring tools are being used more frequently in the neonatal intensive care unit (NICU) to assess brain health. Near infrared spectroscopy (NIRS) is a non-invasive tool to continuously measure regional tissue oxygenation at the bedside. It can be useful in critically ill neonates as a trend monitor to evaluate the balance between tissue oxygen delivery and consumption, providing cerebral and somatic oximetry values, and allowing earlier identification of hemodynamic changes and brain perfusion abnormalities (1, 2). Sensor placement on the forehead easily allows for measurement of regional cerebral oxygen saturation (rScO_2_) and values have been validated with jugular venous saturations in neonates (3, 4). Interpretation of rScO_2_ values must be taken into context with other variables that may affect the cerebral blood flow and oxygenation such as systemic oxygenation (SpO_2_), cardiac output, degree of anemia, carbon dioxide (CO_2_) tension, glucose levels, and metabolic demand. A large multicenter study of preterm infants established cerebral saturation measures between 55 and 85% to be within 2 standard deviations of a median value of 71% (5). Cerebral fractional tissue oxygen extraction (cFTOE) is another useful measure of brain metabolism that reflects the balance between oxygen supply and oxygen consumption and can be calculated by the equation cFTOE = (SpO2 – rScO_2_) / (SpO_2_) (6, 7). Renal regional oxygen saturation (rSrO_2_) values measured by placement of sensor on the posterior flank below the costal margin and above the iliac crest are usually 10–15% higher than cerebral saturations and are sensitive to compromise of systemic blood flow (8).

Amplitude-integrated electroencephalography (aEEG) is a method for continuous monitoring of cerebral function at the bedside (9, 10). aEEG displays both a limited-channel EEG recording and a compressed aEEG tracing that allows evaluation of cerebral background activity pattern over time and facilitates screening for seizures. While EEG remains the gold standard for seizure detection, seizures can reliably be diagnosed by an experienced reader by review of both the compressed aEEG trace and raw EEG signal. However, as aEEG records from a limited number of channels (usually central or parietal), seizures arising from other areas of the brain may not be detected. In addition, brief or low amplitude seizures may be difficult to identify on the compressed tracing. Newer aEEG devices have seizure detection software, which may facilitate seizure detection (2, 9). The use of two channel EEG has improved seizure detection accuracy, and persistent pathological background activity has been associated with poor neurodevelopmental outcome in infants with hypoxic-ischemic encephalopathy (HIE) and in the preterm population (9, 10). Simultaneous use of both NIRS and aEEG may allow better understanding of alterations in hemodynamics and risk of cerebral injury. Several studies have described correlations between aEEG and NIRS monitoring, especially in infants with HIE, but few describe its utility in other clinical scenarios (11, 12). We describe four cases depicting the utility of dual monitoring with aEEG and NIRS in the neonatal population. We then provide a narrative review of existing literature on simultaneous aEEG and NIRS monitoring in the neonate.

## Case Presentation

### Case 1—Hypoxic Ischemic Encephalopathy (HIE)

**Figure 1:**
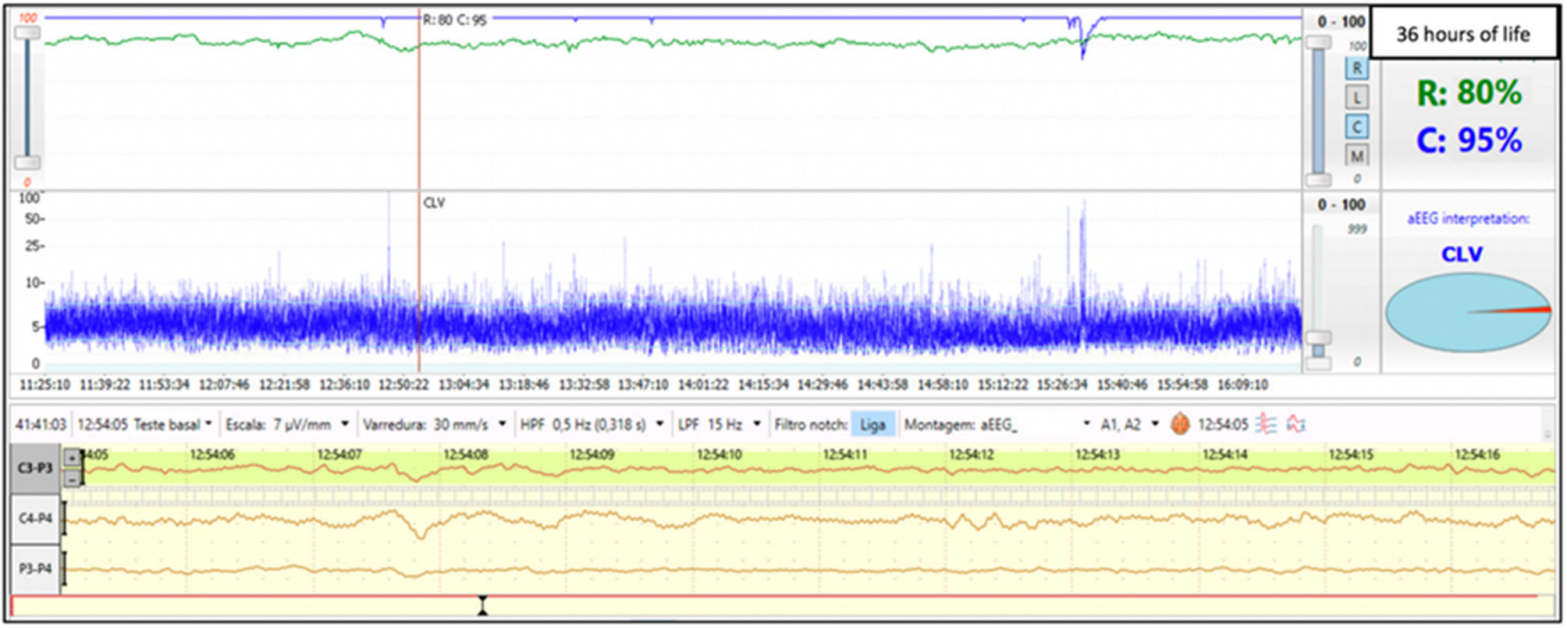
Brain monitoring (aEEG and NIRS) in a term infant undergoing cooling for severe HIE (Sarnat stage III) shows a supranormal rScO_2_ of ~95% while aEEG displays a continuous low voltage pattern.

Term infant born via crash cesarean section due to cord prolapse. Laboratory values were significant for metabolic acidosis with serum pH of 6.9 and base deficit of −21. The infant had severe encephalopathy (Sarnat stage III) on neurological exam performed at 2 h of life and therapeutic hypothermia was initiated. Electrographic seizures were noted at 8 h of life. aEEG monitoring began at 3 h of life and the background activity was continuous low voltage. Initial rScO_2_ was 75%; however, at ~24 h of life, there was an abrupt and sustained increase of rScO_2_ to 95%. This increased cerebral saturation may be explained as a result of secondary energy failure shortly after the primary injury. Reduced consumption of oxygen by injured cerebral tissue leads to a pattern of elevated rScO_2_. Abnormally high cerebral saturation and abnormal aEEG background pattern persisted throughout cooling (Figure 1). Brain MRI was performed on day 7 of life and revealed moderate to severe injury to the thalamus and basal ganglia. Persistence of an abnormal aEEG background pattern for 48 to 72 h and elevated rScO_2_ values at 24 h of life have both been associated with adverse neurodevelopmental outcomes and abnormal neuroimaging (9, 13, 14).

### Case 2—Hemodynamic Instability

**Figure 2:**
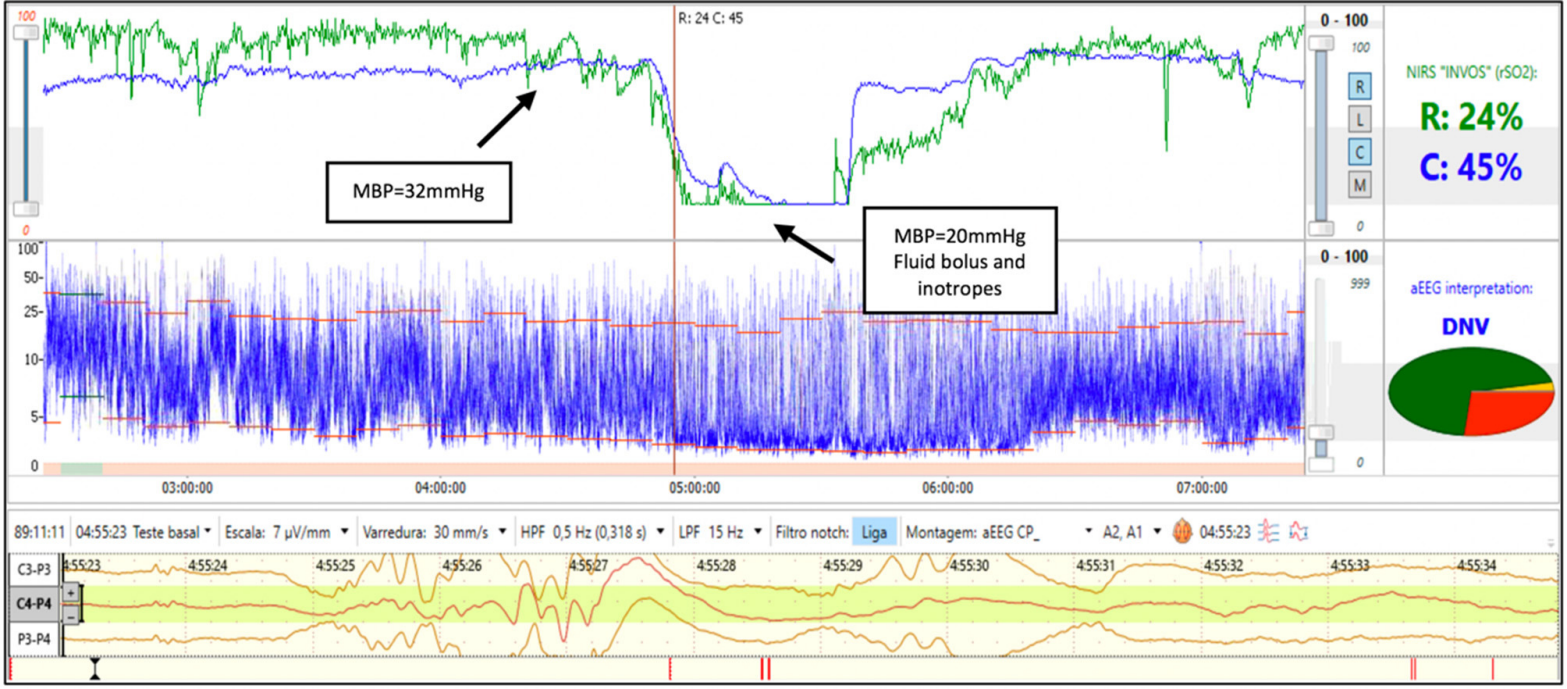
Brain monitoring (aEEG and NIRS) in a preterm infant with septic shock. In this infant with hemodynamic instability an early decrease in rSrO_2_ with normal mean blood pressure (32 mmHg) is seen followed by a decrease in rScO_2_ and associated aEEG burst-suppression (BS) and low blood pressure. After fluid bolus and inotropes, a recovery of rScO_2_ is noted and associated with recovery of background activity to discontinuous normal voltage (DNV).

A 25-week gestation infant with birth weight of 610 g was diagnosed with early onset sepsis and intubated due to respiratory distress syndrome (RDS). During the 3rd day of life the infant developed severe hypotension and septic shock. The rScO_2_ fell from 75 to 15% and simultaneously the aEEG background activity changed from discontinuous normal voltage to burst suppression. Two site NIRS was used and a ~30% reduction in rSrO_2_ was noted while the mean arterial blood pressure remained in the normal range for gestational age. The decrease in renal saturation preceded cerebral desaturation by 40 min. After a fluid bolus and initiation of inotropes, the rScO_2_ and rSrO_2_ increased and was followed by a recovery in aEEG background activity to discontinuous normal voltage (Figure 2). Somatic desaturation is often an early indicator of shock and ensuing cerebral desaturation and alterations in cerebral activity may persist until hemodynamic stability is restored.

### Case 3—Patent Ductus Arteriosus (PDA)

**Figure 3:**
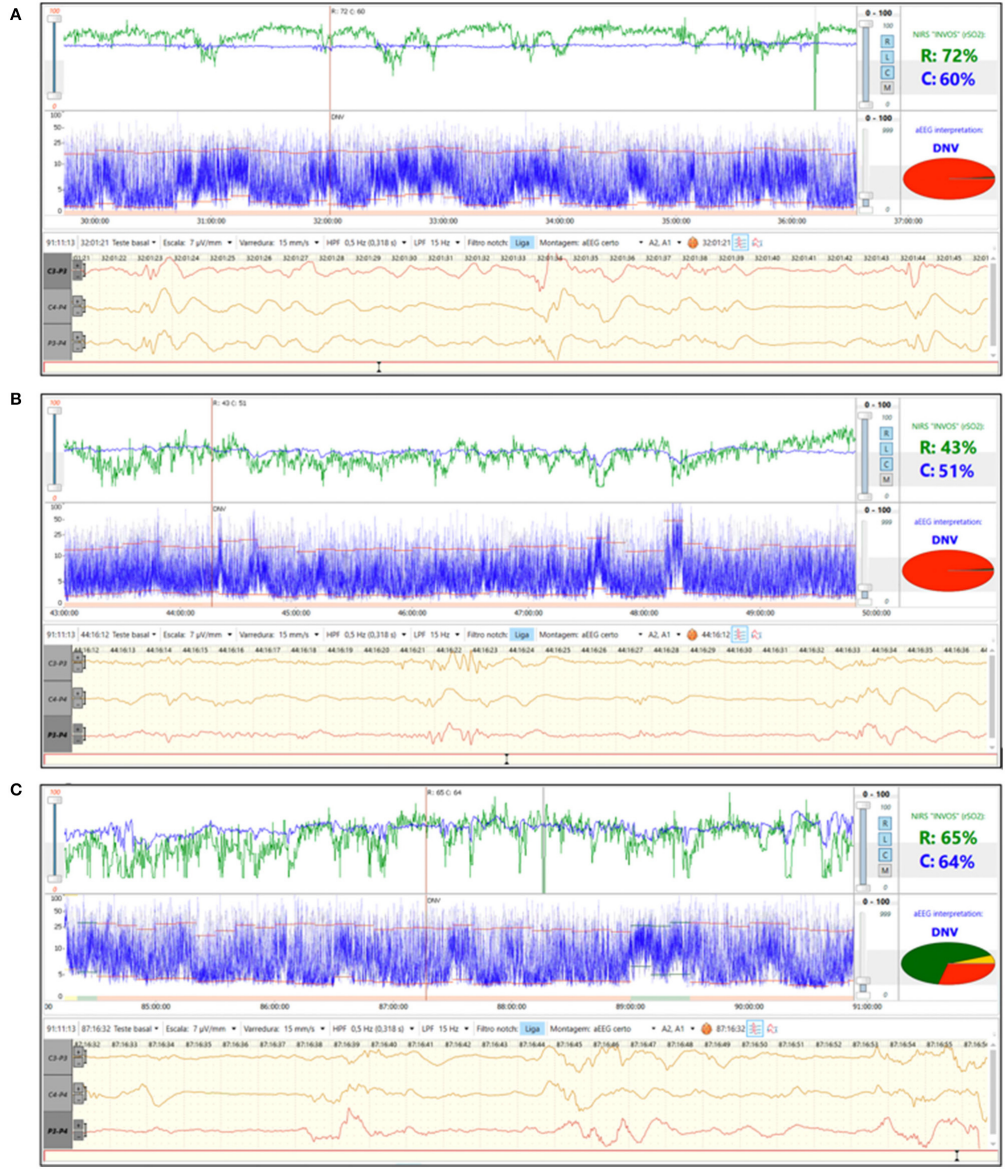
**(A)** Brain monitoring (aEEG and NIRS) in a preterm baby on 2nd day of life. aEEG background pattern was discontinuous normal voltage with immature SWC and rScO_2_~65%. **(B)** Brain monitoring (aEEG and NIRS) on 3rd day of life. hsPDA was diagnosed and associated with decrease in rScO_2_ and loss of SWC. **(C)** Brain monitoring (aEEG and NIRS) on 5th day of life. At this time the PDA was found to be closed by echocardiogram. There was return of rScO_2_ to normal range and a return of immature SWC on aEEG.

A 27-week gestation infant with birth weight 945 g was diagnosed with RDS and was clinically stable on continuous positive airway pressure (CPAP) after birth. During the 2nd day of life aEEG background pattern was discontinuous normal voltage with immature sleep wake cycles (SWC) and rScO2~65% (Figure 3A). On day of life 3 there was a decrease in both cerebral and renal saturation (rScO_2_~50%, rSrO2~40%) together with loss of SWC (Figure 3B). Echocardiogram was performed and diagnosed a hemodynamically significant patent ductus arteriosus (hsPDA). Indomethacin was given. On the 5th day of life, rScO_2_ increased to the normal range and this change was associated with return of immature SWC (Figure 3C). PDA closure on echocardiogram was confirmed. An hsPDA is associated with increased pulmonary blood flow and decreased systemic blood flow causing low cerebral and renal saturations due to ductal steal. Simultaneous monitoring of aEEG with loss of immature SWC suggests that PDA-related changes in oxygenation also impact cerebral function.

### Case 4—Seizures

**Figure 4:**
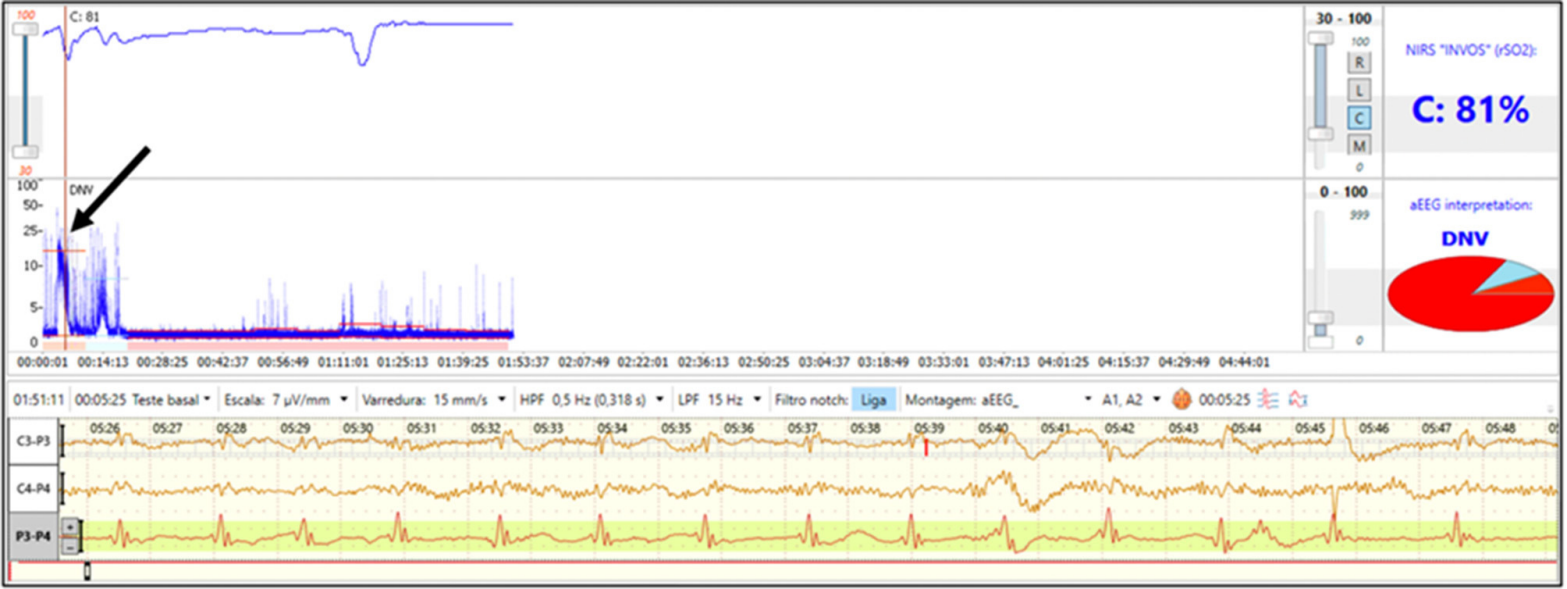
Brain monitoring (aEEG and NIRS) in a preterm infant with severe anemia after placental abruption who had clinical seizures on 1st day of life. The aEEG demonstrates a flat tracing with seizure activity (arrow). Simultaneous NIRS tracing shows supranormal rScO_2_ which transiently decrease during seizure activity.

A 30-week preterm infant born via emergency cesarean section due to severe maternal bleeding was diagnosed with severe anemia (hematocrit 15%) and neonatal encephalopathy. Immediate transfusion of packed red blood cells was performed and NIRS was initiated after the blood transfusion. The initial aEEG shows flat trace with seizures (Figure 4) and the rScO_2_ is above 90%. During seizures, a drop in rScO_2_ was noted. As NIRS values reflect the balance of oxygen delivery and regional consumption, a decline in rScO_2_ during seizures may be explained by the increased cerebral metabolic demand occurring with seizure activity.

## Discussion

### Assessing Brain Oxygenation and Brain Function Using Simultaneous NIRS and aEEG Nomenclature

The use of aEEG for brain monitoring has been well-established in the context of HIE and cooling. The normal patterns for term and preterm infants have also been studied (16). Similarly cerebral NIRS monitoring in the first 3 days of life was studied in a large European multicenter randomized trial (SafeBoosC II—Safeguarding the Brains of our smallest Children). Results from this study successfully demonstrated the use of cerebral oxygenation monitoring to reduce the hypoxic and/or hyperoxic burden on the preterm brain (17). As NIRS and aEEG have separately been investigated in previous clinical trials such as these, the two technologies are routinely used in NICUs worldwide; however, the combined use of these brain monitoring techniques has had limited investigation. These bedside neuromonitoring devices are easily employed in the NICU and have the potential to provide complementary information regarding cerebral hemodynamics and cerebral electrical activity.

Several small cohort studies in infants with HIE found abnormal aEEG background and/or abnormally high cerebral NIRS values to be associated with adverse outcomes as we describe in Case 1. Ancora et al. (12) studied 12 neonates undergoing cooling and concluded that early aEEG background abnormalities at 12 h did not have a high positive predictive value (PPV) for abnormal outcome (PPV 36.4%), while cerebral saturation starting at 12 h was found to be significantly higher in infants with a poor outcome when compared to those with a normal outcome. High cerebral saturation was felt to reflect luxury reperfusion following a severe cerebral injury. Secondary energy failure is evident with a reduction in oxygen consumption by the severely injured neuronal cells. In contrast, in a cohort of 39 newborns with HIE, Niezen et al. (15) found that an early severely abnormal aEEG predicted abnormal outcome as did epileptic activity, while high rScO_2_ was not associated with adverse outcome until 72 h of age and later. Lemmers et al. (18) studied the prognostic value of aEEG and NIRS in 39 term newborns with HIE. rScO_2_ was significantly higher and cerebral fractional tissue oxygen extraction (cFTOE) lower in the group with abnormal outcome beginning at 24 h of age. aEEG scores in both groups improved during hypothermia with significantly higher scores in the group with a favorable outcome. Both aEEG and rScO_2_ were early predictors of favorable outcome, but less reliable for predicting adverse outcome. The authors showed that compared to either modality alone, combining NIRS and aEEG data improved the PPV (NIRS 67% and aEEG 62% vs. combined 91%) and negative predictive value (NPV) (73 and 100% vs. 100%) at 12 to 18 h of age. Meta-analyses of studies predicting outcomes in HIE using aEEG have concluded that the maximum prediction is achieved at 72 h and the background patterns that most accurately predict outcome are burst suppression, continuous low voltage, and flat tracing (9, 14).

NIRS monitoring has been used extensively for assessment of hemodynamic status in preterm infants and to quantify end organ blood flow. In the setting of shock, somatic tissue oxygenation as measured by NIRS may be compromised first, particularly if cerebral autoregulation remains intact. The early impact on somatic tissue oxygenation has also been documented in neonates with congenital heart disease (19) and in children with dehydration requiring volume resuscitation (20) due to redistribution of blood flow with hemodynamic compromise. NIRS monitoring to guide management of hypotension in preterm infants (21) found a reduction in the burden of hypotension. In contrast, there has been limited use of aEEG in patients with hemodynamic instability. One previous study demonstrated an association between hemodynamic instability and aEEG abnormalities, finding that infants on inotropes had significantly lower aEEG amplitude and continuity (22). Infants in early shock may have reduction of regional tissue oxygenation and pathological aEEG background activity as shown in Case 2, even prior to significant hypotension. Clinical interventions to improve blood pressure and perfusion may lead to recovery of normal tissue oxygenation values and aEEG background activity. The combined use of aEEG and NIRS may further guide timing and need for interventions in infants at risk for hemodynamic instability.

Preterm infants with a hsPDA (as in Case 3) may also benefit from combined neuromonitoring. The left-to-right shunt causes a ductal steal phenomenon and may adversely affect both perfusion and oxygenation to the brain and kidney. Preterm infants with a hsPDA may have lower cerebral and renal saturations and a higher risk for IVH and white matter injury. A previous study conducted by Chock et al. concluded that decreased renal saturation (rSrO_2_ <66%) was associated with the presence of an hsPDA (23). Other studies have demonstrated a significant increase in both cerebral and renal saturation after either medical or surgical PDA closure (24, 25). Diminished cerebral perfusion in the presence of a PDA may further lead to changes in cerebral function. Limited studies have found a significant PDA in preterm infants to be negatively associated with aEEG scores and continuity (26, 27). Lemmers et al. (28) published the only study using both NIRS and aEEG monitoring together in preterm infants with PDA. In their study of 20 preterm infants undergoing ductal ligation, 65% decreased their rScO_2_ accompanied by a decrease in aEEG amplitudes. The effect on aEEG amplitude was most notable in the babies with the lowest rScO_2_ values and is similar to what was described in Case 3.

The effect of seizure activity on cerebral NIRS values as described in Case 4 demonstrates the important coupling between oxygen delivery and cerebral metabolic demand. Several investigators have also studied the relationship between electrocerebral activity as measured by aEEG and measures of oxygen delivery and consumption as measured by NIRS. Caicedo et al. (29) studied a cohort of 22 infants undergoing sedation with propofol. They compared how the NIRS variables, rScO_2_ and cFTOE, were related to aEEG measures such as inter-burst interval and amplitude. The higher values of FTOE were related to higher values of EEG amplitude. This suggested that higher extraction of oxygen is needed to support brain metabolism. Ter Horst et al. (6) investigated the relationship between rScO_2_, cFTOE and aEEG measures in 46 preterm infants. aEEG amplitude changed with increasing gestational age while cFTOE and aEEG amplitude also increased with postnatal age. The authors concluded that there is a significant relationship between electrocortical activity and oxygen consumption as cFTOE increases with increasing aEEG amplitude. The combination of high cFTOE and low electrocortical activity is likely to reflect decreased cerebral oxygen delivery or cerebral blood flow and should alert the clinician to the risk for brain injury. Wallois et al. (30) reported the advantages of the combined use of NIRS and video EEG for continuous monitoring allowing access to the time course of hemodynamic changes during different stages of seizures. Combined monitoring could be used for classification of seizures according to hemodynamic responses. Based on the level of cerebral deoxygenation during seizures, NIRS values might provide predictive criteria for risk and also for assessment of the impact of therapies introduced in the management of seizures. Moreover, hemodynamic abnormalities observed in the preictal phase could be associated and used as a predictor for seizures in the future.

Dual monitoring in several other clinical scenarios has been published. One such condition is for prediction of IVH. A large (*n* = 127) observational study of preterm infants <32 weeks' gestation was performed to determine if the risk for IVH and death in the first 72 h of life could be predicted using NIRS and aEEG monitoring (31). Infants who developed severe IVH or death had significantly lower rScO_2_ from 8 to 10 min of life. aEEG was not predictive of death or IVH; however, the aEEG device used had artifacts caused by resuscitation and handling which likely limited its interpretation.

Several studies have also focused on simultaneous NIRS and aEEG monitoring in term and preterm infants in the delivery room. An observational study of aEEG and cerebral NIRS in newborns ≥34 weeks' gestation was performed with the goal of determining its feasibility and whether cerebral activity of babies requiring resuscitation differed from those with an uncomplicated neonatal transition (32). Infants not requiring delivery room resuscitation had initially higher minimum and maximum aEEG amplitudes followed by a decrease at 4 to 5 min of age. Infants requiring respiratory support had significantly lower SpO_2_ and rScO_2_ from minute 4 to 8 indicating reduced oxygen delivery. A subsequent observational study using the same study design found that minimum and maximum amplitude were lower in the group requiring resuscitation during the 15-min study period. Cerebral saturation was significantly lower until 11 min and cFTOE was significantly higher until 10 min while SpO_2_ was within normal ranges in both groups (33). The authors concluded that neonates requiring resuscitation after birth had a lower cerebral saturation and concurrently lower cerebral activity but increased cFTOE. They speculated that cerebral monitoring with NIRS and aEEG may provide useful information during neonatal resuscitation.

As neurocritical care has become a significant focus in the neonatal intensive care setting, both aEEG and NIRS have shown utility as bedside monitors in a population of babies at high risk for brain injury. While established individually as diagnostic and prognostic techniques, there are limited studies describing their combined use to evaluate both brain function and brain oxygenation. These four clinical scenarios demonstrate how simultaneous monitoring of aEEG and NIRS provides important clinical information, particularly in neonates with HIE, shock, an hsPDA, and seizures. Changes in brain activity as seen on aEEG often follow abnormal NIRS values, and the impact of a hemodynamic disturbance on the brain can be assessed by the ensuing alteration of aEEG patterns. We speculate that routine combination of these monitoring modalities may become the future standard for neonatal neuromonitoring. Further investigation is necessary to evaluate clinical situations in which the combined use of these devices is recommended over use of each one separately.

## Data Availability Statement

The datasets generated for this study are available on request to the corresponding author.

## Ethics Statement

This study was carried out in accordance with the recommendations of Declaration of Helsinki, Santa Casa de Misericordia Committee. The protocol was approved by the Santa Casa de Misericordia Committee. Written informed consent was obtained from parents/guardian for the publication of this case report.

## Author Contributions

GV has made substantial contributions to the conception, design and draft of the work, approved the submitted version (and any substantially modified version that involves the author's contribution to the study) and agreed both to be personally accountable for the author's own contributions and to ensure that questions related to the accuracy or integrity of any part of the work, even ones in which the author was not personally involved, are appropriately investigated, resolved, and the resolution documented in the literature. VC and KV have made substantial contributions to conception, design and revision, approved the submitted version (and any substantially modified version that involves the author's contribution to the study) and agreed both to be personally accountable for the author's own contributions and to ensure that questions related to the accuracy or integrity of any part of the work, even ones in which the author was not personally involved, are appropriately investigated, resolved, and the resolution documented in the literature. AN and RP have made substantial contributions to acquisition and analysis of the data, approved the submitted version (and any substantially modified version that involves the author's contribution to the study) and agreed both to be personally accountable for the author's own contributions and to ensure that questions related to the accuracy or integrity of any part of the work, even ones in which the author was not personally involved, are appropriately investigated, resolved, and the resolution documented in the literature.

### Conflict of Interest

The authors declare that the research was conducted in the absence of any commercial or financial relationships that could be construed as a potential conflict of interest.
